# Effect of pre-weaning concentrate supplementation on peripheral distribution of leukocytes, functional activity of neutrophils, acute phase protein and behavioural responses of abruptly weaned and housed beef calves

**DOI:** 10.1186/1746-6148-8-1

**Published:** 2012-01-04

**Authors:** Eilish M Lynch, Mark McGee, Sean Doyle, Bernadette Earley

**Affiliations:** 1 Animal and Bioscience Research Department, Animal & Grassland Research and Innovation Centre, Teagasc, Grange, Dunsany, Co. Meath, Ireland; 2 Department of Biology and National Institute for Cellular Biotechnology, National University of Ireland Maynooth, Co. Kildare, Ireland; 3 Livestock Systems Research Department, Animal & Grassland Research and Innovation Centre, Teagasc, Grange, Dunsany, Co. Meath, Ireland

## Abstract

**Background:**

The effect of pre-weaning concentrate supplementation on peripheral distribution of leukocytes, functional activity of neutrophils, acute phase protein response, metabolic and behavioural response, and performance of abruptly weaned and housed beef calves was investigated. Calves were grazed with their dams until the end of the grazing season when they were weaned and housed (day (d) 0) in a concrete slatted floor shed, and offered grass silage *ad libitum *plus supplementary concentrates. Twenty-six days prior to weaning and housing, 20 singled suckled, pure-bred Simmental male (non-castrated), (n = 10, m) and female (n = 10, f) calves were assigned to one of two treatments (i) concentrate supplement (CS: n = 10 (5 m and 5 f), mean age (s.d.) 201 (12.8) d, mean weight (s.d.) 258 (20.2) kg) or (ii) no concentrate supplement (controls) (NCS: n = 10, (5 m and 5 f), mean age (s.d.) 201 (13.4) d, mean weight (s.d.) 257 (19.6) kg) pre-weaning.

**Results:**

There was a treatment × sampling time interaction (*P *< 0.05) for percentage CD4^+ ^and WC1^+ ^(γδ T cells) lymphocytes and concentration of plasma globulin. On d 2, percentage CD4^+ ^lymphocytes decreased (*P *< 0.001) in both treatments. Subsequently on d 7, percentage of CD4^+ ^lymphocytes increased (*P *< 0.01) in CS compared with d 0, whereas percentage of CD4^+ ^lymphocytes in NCS did not differ (*P *> 0.05) from d 0. On d 2, WC1^+ ^lymphocytes decreased (*P *< 0.05) in both treatments but the decrease was greater (*P <*0.05) in NCS than CS. Subsequently, percentages did not differ (*P *> 0.05) from pre-weaning baseline. On d 2, the increase in concentration of globulin was greater (*P *< 0.05) in CS compared with NCS, and subsequently there was no difference between treatments. Pre-weaning ADG was 1.07 (s.e.m.) (0.26) kg and 0.99 (s.e.m.) (0.26) kg for CS and NCS, respectively. Post-weaning, CS calves spent more time lying compared with NCS calves.

**Conclusions:**

Calves supplemented with concentrate prior to weaning had a lesser reduction in WC1^+ ^lymphocytes, increased percentage CD4^+ ^lymphocytes and concentration of total protein, and spent more time lying post-weaning, compared with non-supplemented calves.

## Background

Within seasonal, grassland-based suckler beef production systems in Ireland, calves are generally spring-born and reared with their dam at pasture for approximately 8 months until the end of the grazing season in autumn when they are weaned. At, or, shortly after weaning, calves are housed indoors over the winter period and offered grass silage, which is generally supplemented with concentrates [1]. Concentrate supplementation of suckling, grazing beef calves prior to weaning is commonly referred to as 'creep feeding', and serves to compensate for decreasing milk yield and forage, and to improve calf weaning weights [2-6]. Additionally, this practice is often advocated as a means of reducing weaning stress in calves through the familiarisation to a palatable feed, such as concentrates [7] and has been reported to decrease morbidity in feedlots [6].

Weaning is a stressful event in the calf's lifetime with alterations in behaviour [8-10], hormonal mediators of stress [11,12] and immune function [13-16] evident post-weaning. Deferring housing at the time of weaning results in a less marked stress response compared with the traditional practice of weaning and simultaneous housing [17]. Previous studies have also examined the effects of pre-weaning concentrates on measures of post-weaning physiological and immunological responses [14]. Abrupt weaning coupled with housing causes transitory neutrophilia, mediated by reduced surface expression of L-selectin, and transient lymphopaenia, characterised by decreased percentage CD4^+^, CD8^+ ^and WC1^+ ^lymphocytes and increased MHC class II^+ ^cells [15]. Although, some studies have examined the effects of pre-weaning calf management, such as two-stage weaning with nose-clips [18,19], on the stress response of beef calves post-weaning.

Thus, the objectives of the present study were to examine the effects of offering concentrate supplementation to beef calves (CS) for 26 days prior to abrupt weaning and housing on (i) peripheral distribution of leukocytes, (ii) functional activities of neutrophils, and (iii) acute phase protein response compared with calves that were not offered concentrate supplementation (NCS) prior to weaning. The metabolic and behavioural responses of calves were also characterised to provide supplementary information.

## Methods

All animal procedures performed in this study were conducted under experimental licence from the Irish Department of Health and Children in accordance with the Cruelty to Animals Act 1876 and the European Communities (Amendment of Cruelty to Animals Act 1876) Regulation 2002 and 2005.

### Animal management and experimental design

Twenty, spring-born (mean date of birth (s.d.) 23 March (12.7) d), singled suckled, pure-bred Simmental male (non-castrated, n = 10) and female (n = 10) calves were used in this study. Calves and their dams were rotationally grazed together on a predominantly perennial ryegrass (*Lolium perenne*)-based sward from April until housing in a concrete slatted floor shed at the end of the grazing season (4 November). Twenty-six days prior to weaning and housing (d -26), calves were weighed, vaccinated subcutaneously against bovine respiratory syncytial virus, bovine parainfluenza-3 virus, bovine viral diarrhoea virus, and infectious bovine rhinotracheitis virus using Rispoval-3 and Rispoval IBR (Pfizer Animal Health, Ireland). Then, within gender, calves were blocked according to weight and age and assigned to one of two treatments (i) concentrate supplementation offered (CS: n = 10, mean age (s.d.) 201 (12.8) d, mean weight (s.d.) 258 (20.2) kg, 5 males and 5 females) or (ii) no concentrate supplementation offered (controls) (NCS: n = 10, mean age (s.d.) 201 (13.4) d, mean weight (s.d.) 257 (19.6) kg, 5 male and 5 female) during a pre-weaning period (d -26 to d 0). During the pre-weaning period (d -26 to d 0), the CS and NCS calves and their respective dams were grazed as two separate groups in adjacent paddocks of similar herbage mass (~1250 kg dry matter (DM)/ha pre-grazing) and nutritive value (205 g/kg DM crude protein (CP), 472 g/kg DM neutral detergent fibre (NDF), 792 g/kg DM *in vitro *organic matter digestibility (OMD)). All animals were habituated to close visual inspection by the stockperson daily. The CS calves were offered concentrates (430 g/kg rolled barley, 430 g/kg beet pulp, 80 g/kg soyabean meal, 45 g/kg molasses, 15 g/kg mineral/vitamin premix, 116 g/kg DM CP, 223 g/kg DM NDF, 863 g/kg DM *in vitro *OMD) in a metal feeding trough once daily. The allowance of concentrate was increased in increments of 0.25 kg daily until 1.0 kg of concentrates per animal daily was attained. To permit feeding the concentrate supplement, calves were temporarily separated from the cows. The feeding trough was of sufficient length for all calves to eat simultaneously, and all calves participated in the consumption.

**Figure 1:**
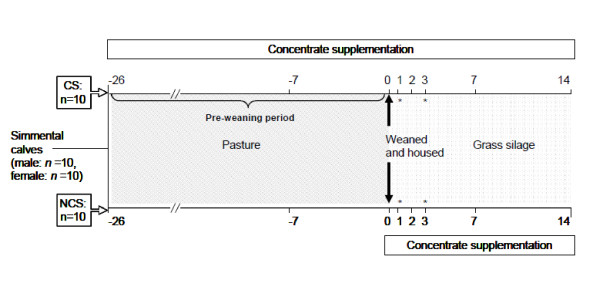
**Experimental design and sample collection schedule for beef calves that were supplemented with concentrates for 26 d prior to abrupt weaning (d 0) and subsequently offered grass silage plus concentrates at housing**. * denotes additional blood collection time-points for haematological analysis and rectal body temperature recordings. Concentrate supplemented (CS) calves were offered concentrates for 26 d prior to weaning, whereas non supplemented (NCS) calves were not offered concentrates during the pre-weaning period (d -26 to d 0). On d 0, all calves were abruptly weaned, housed and offered grass silage *ad libitium *plus concentrates.

On d 0, calves were abruptly weaned from their dams and housed in 4 pens (containing either 5 male or 5 female calves per pen) according to previous treatments in a concrete slatted floor shed at a space allowance of 3.7 m^2^/head. They were offered grass silage (687 g/kg *in vitro *DM digestibility) *ad libitum *plus supplementary concentrates (above formulation). The CS calves continued to receive 1.0 kg of concentrates per head daily, whereas NCS calves were offered 0.25 kg of concentrates until 1.0 kg of concentrates per head daily was reached (Figure 1). Cows were relocated having no audio or visual contact with their calves.

### Rectal body temperature and live weight

Rectal body temperature of calves was recorded before blood sampling on d -7, 0 (housing), 1, 2, 3, 7, and 14 using a digital thermometer (Jørgen Kruuse; Marslev, Denmark). Live weight was recorded on d -26, d 0 and d 14.

### Sample collection

Blood was collected into vacutainers (Vacuette, Cruinn Diagnostics, Ireland) containing the appropriate anticoagulant via direct jugular venipuncture using mild restraint in a holding chute at d -7, 0 (weaning), 1, 2, 3, 7 and 14 (Figure 1). Blood samples were transported to the laboratory, stored at ambient temperature and processed within 3.5 h.

### Total and differential leukocyte populations

Total leukocyte, neutrophil, lymphocyte, monocyte, eosinophil, and basophil were determined from K_3_EDTA anti-coagulated blood (6 mL) using an automated haematology analyzer (ADVIA 2120, Bayer Healthcare, Siemens, UK) equipped with software for bovine blood.

### Leukocyte immunostaining

Acid citrate dextrose anti-coagulated blood (8 mL) was used for leukocyte immunostaining using a whole blood assay [20,21]. Briefly, duplicate 1 mL aliquots of whole blood were transferred to a 5 mL test tube (Sarstedt, Nümbreacht, Germany) and tubes were centrifuged at 250 × *g *for 5 min at 4°C. After aspiration of supernatants, 3 mL of BD FACS lysing solution (BD Biosciences, Oxford, UK) was added for 10 min at room temperature to lyse erythrocytes. Remaining leukocytes were resuspended in 1.5 mL of sheath fluid (Coulter Isoton II Diluent (Beckman Coulter UK Ltd., High Wycombe, UK) and counted using a Z1 Coulter Particle Counter (LABPLAN Ltd., Galway, Ireland). One hundred microliter aliquots of cell suspension (1 × 10^6 ^cells) were seeded into series of wells on a 96-well microtiter plate. Leukocyte immunostaining was carried out using a 2-antibody system, as described by Weber *et al*. 2001 [20], with the exception of the secondary (detection) antibody where FITC-labelled goat anti-mouse IgG F(ab')2 (Southern Biotech, Birmingham, AL, USA) was used following a 1/100 dilution with PBS-0.01 (w/v) % BSA. Sources, specificities, isotypes and working solutions of monoclonal and secondary antibodies are described in Table 1. All antibodies were diluted to final working concentration using PBS-0.01% (w/v) BSA (pH 7.2). Following this procedure, cells were fixed with 200 μl of 1% (v/v) paraformaldehyde, and further diluted with 800 μl of sheath fluid for immediate acquisition on a Partec CyFlow SL flow cytometer (Partec, Münster, Germany). Cells were kept at 4°C in the dark prior to acquisition.

**Table 1:** Antibodies used in the immunostaining of leukocyte surface markers


**Specificity**	**Cell types**	**Clone**	**Isotype**	**Working solution**	**Source^1^**

CD4	T-helper/inducer cells	CC8	IgG_2_a	7 μL/mL	Serotec
CD8	T-cytotoxic/suppressor cells	CC63	IgG_2_a	7 μL/mL	Serotec
WC1	Subset of γδ T cells	CC15	IgG_2_a	7 μL/mL	Serotec
MHC class II	Antigen presenting cells (B cells, activated T cells)	H42A	IgG_2_a	7 μL/mL	VMRD
CD62L	L-selectin	BAQ92A	IgG_1_	14 μL/mL	VMRD
G1	Neutrophils (and eosinophils)	MM20A	IgG_1_	14 μL/mL	VMRD
CD45	All leukocytes (pan marker)^2^	CC1	IgG_1_	3.5 μL/mL	Serotec
IgG_1 _isotype	Mouse IgG_1_a negative control	-	-	7 μL/mL	Serotec
IgG_2_a isotype	Mouse IgG_2_a negative control	-	-	7 μL/mL	Serotec

^1^Serotec (Oxford, UK), VMRD (Pullman, WA, USA).

^2^Used to differentiate leukocyte populations and exclude debris (gate analysis).

### Phagocytic and oxidative burst activity assays

The phagocytic and oxidative burst activity of neutrophils was determined in lithium heparin anti-coagulated blood (8 mL) using the Phagotest kit and Bursttest (PhagoBurst) kit (Orpegen Pharma, Heidelberg, Germany) following the manufacturer's instructions, with modifications [22], on a Partec CyFlow SL flow cytometer (Partec Gmbh, Münster, Germany). Duplicate tests for each sample were performed.

### Flow cytometric analysis

#### Immunophenotypes

A minimum of 30,000 events were acquired and analyzed using FloMax software (Partec GmbH, Münster, Germany). Lymphocytes and neutrophils were gated from all other leukocyte populations based on their forward and side scatter characteristics on dot plots and were confirmed using CD45 (pan leukocyte marker) staining [23]. The percentage lymphocytes staining positive for CD4, CD8, WC1+ (γδ T cells), and MHC class II, and percentage of neutrophils staining positive for G1 was recorded. Surface expression of CD62L was recorded as mean fluorescence intensity (MFI) of CD62L-positive staining neutrophils. A threshold for positive staining cells was set using histograms identifying irrelevant isotype controls and PBS-0.01 (w/v) % BSA only treated leukocytes.

#### Phagocytic and oxidative burst activity assays

Data were acquired from 15,000 cells per sample and analysis was carried out using FloMax software (Partec Gmbh, Münster, Germany). For each sample, the percentage phagocytosis positive and oxidative burst positive cells within the neutrophil gate were recorded.

### Acute phase protein response (fibrinogen and haptoglobin)

Blood collected into vacutainer tubes containing lithium heparin (8 mL) and sodium citrate (4.5 mL) was used to determine the concentration of haptoglobin and fibrinogen, respectively. Plasma was harvested following centrifugation at 1600 × *g *at 4°C for 15 min and stored at -80°C until assayed. Concentration of plasma haptoglobin was measured using an automatic analyzer (spACE, Alfa Wassermann, Inc., West Caldwell, NJ, USA) and commercial assay kit (Tridelta Development Ltd., Wicklow, Ireland) according to the manufacturer's procedure as described by [24]. Concentration of fibrinogen was measured using an automatic analyzer (spACE, Alfa Wassermann, Inc., West Caldwell, NJ, USA) using a previously described method [25].

### Metabolite analysis

Blood collected into vacutainer tubes containing lithium heparin (8 mL) and sodium citrate (4.5 mL) was used to determine the concentration of metabolic variables on d -7, 0 (weaning), 2, 7 and 14. Plasma was harvested following centrifugation at 1600 × *g *at 4°C for 15 min and stored at -80°C until assayed. Concentration of plasma albumin, globulin, total protein, creatine kinase, non-esterified fatty acids (NEFA), glucose and β-hydroxybutyrate (βHB) were analysed on an automatic analyser (Olympus AU400, Japan) using reagents supplied by Olympus

### Behaviour

Lying and walking behaviour was monitored using IceTag motion sensor pedometers (IceTag 2.004, IceRobotics, Midlothian, Scotland, UK) attached to the front left leg of each calf with a Velcro strap on d 0 (weaning and housing), and were removed on d 14. Data from IceTag pedometers were downloaded onto a PC with the software IceTag AnalyserTM (2.009), and steps per day, percentage of time spent lying, standing or active were registered. Seven complete days of data were used in the analysis.

Feeding behaviour was monitored using 24 h continuous camera recordings of each pen. Cameras were connected to a DVD recorder via a multi-vision system (Robot, duplex multiplexer), which allowed pictures from all cameras to be viewed on one screen at one time, and were calibrated with time and date settings. For each pen, behavioural observations were recorded on d 0 (weaning and housing), 1, 2, 3, 7 and 14. The calves were observed by instantaneous scan sampling and the interval between scans was 10 min. Each pure-bred Simmental calf was identified by body markings and observed for eating concentrates (head in the concentrate trough), eating silage at the feed face and drinking water. The number of times each behaviour occurred was recorded for each scan time point.

### Statistical Analysis

All statistical analysis was performed using SAS/STAT for Windows (SAS, 2003). Data were tested for normality using PROC UNIVARIATE, and data (eosinophil and basophil number, phagocytosis positive neutrophils MFI, oxidative burst positive neutrophils MFI, βHB, creatine kinase and urea) that did not meet parametric assumptions were log transformed prior to statistical analysis. Data were then analysed as repeated measures using the PROC MIXED procedure of SAS with an unstructured covariance matrix within animal. The effects of treatment, sampling time, treatment × sampling time interaction and gender were listed in the model statement and day -7 was included as a covariate. For behavioural data, no covariate was included in the model. As animals were subjected to continuous recordings for measurement of behaviour, total count data for eating and drinking behaviours was expressed as percentage time. Least squares means were estimated and differences between least squares means were determined using Tukey-Kramer Adjustment for multiple comparisons. A probability of *P <*0.05 was selected as the level of significance.

## Results

### Rectal body temperature

There was no treatment × sampling time interaction (*P *> 0.05) for rectal body temperature. The overall average rectal body temperature was greater (*P *< 0.01) in NCS calves than CS calves (Table 2). On d 1 and 2, rectal temperature increased (*P *< 0.01) but subsequently, did not differ (*P *> 0.05) from pre-weaning baseline (d 0) (Table 2).

**Table 2:** Effect of concentrate supplementation for 26 days pre-weaning on rectal body temperature in abruptly weaned and housed beef calves


		**Days post-weaning**							***P*-values^1^**		
		
		**0**	**1**	**2**	**3**	**7**	**14**	**Pooled s.e.m**.	**T**	**S**	**T×S**

Rectal body temperature, °C	CS	38.3^a^	39.0^b^	38.7^b^	38.5^a^	38.6^a^	38.4^a^	0.11	**	**	NS
	NCS	38.5^a^	39.1^b^	38.9^b^	38.6^a^	38.6^a^	38.6^a^				

T = treatment, S = sampling time, T × S = treatment × sampling time interaction.

CS = concentrate supplement prior to abrupt weaning (*n *= 10).

NCS = no concentrate supplement prior to abrupt weaning (*n *= 10).

^1^Levels of significance: NS = *P *> 0.05, ** = *P *< 0.01. ^a, b^Within a row, least squares means without a common superscript differ (*P <*0.05).

### Total leukocyte and differential number

There was no effect (*P *> 0.05) of treatment or treatment × sampling time interaction for total leukocyte, neutrophil, lymphocyte, monocyte, eosinophil or basophil number or of sampling time for total leukocytes, eosinophils or basophils (Table 3). However, neutrophil number increased (*P *< 0.001) on d 1 and d 2, whereas lymphocyte number decreased (*P *< 0.001) on d 1 and d 2, and subsequently did not differ (*P *< 0.05) compared with pre-weaning baseline. Monocyte number decreased on d 14 compared with pre-weaning baseline.

**Table 3:** Effect of concentrate supplementation for 26 days pre-weaning on total leukocyte and differential populations in abruptly weaned and housed beef calves


		**Days post-weaning**							***P *values^1^**		
		
**Cell type**, **×10^3^cells/μL**		**0**	**1**	**2**	**3**	**7**	**14**	**Pooled s.e.m**.	**T**	**S**	**T×S**

**Total leukocytes**	CS	10.5	11.3	10.4	10.1	10.0	9.3	0.52	NS	NS	NS
	NCS	10.3	10.7	9.6	9.9	9.8	10.0				
**Neutrophils**	CS	2.5^a ^	4.2^b ^	3.7^b^	2.5^a^	2.8^a ^	2.3^a ^	0.31	NS	***	NS
	NCS	2.6^a ^	4.8^b^	3.9^c^	2.7^a^	2.7^a^	2.7^a^				
**Lymphocytes**	CS	7.3^a^	5.2^b^	5.3^b^	6.6^a^	6.7^a^	6.6^a^	0.33	NS	**	NS
	NCS	6.7^a^	4.9^b ^	5.0^b^	6.0^a^	7.1^a^	6.4^a^				
**Monocytes**	CS	0.68^a^	0.61^ab^	0.58^ab^	0.61^ab^	0.63^ab^	0.37^b^	0.063	NS	**	NS
	NCS	0.73	0.69	0.70	0.73	0.82	0.51				
**Eosinophils**	CS	0.21	0.26	0.21	0.17	0.10	0.16	0.048	NS	NS	NS
	NCS	0.22	0.20	0.24	0.26	0.15	0.19				
**Basophils**	CS	0.09	0.11	0.12	0.11	0.08	0.13	0.029	NS	NS	NS
	NCS	0.11	0.10	0.09	0.09	0.08	0.10				

T = treatment, S = sampling time, T × S = treatment × sampling time interaction.

CS = concentrate supplement prior to abrupt weaning (*n *= 10). NCS = no concentrate supplement prior to abrupt weaning (*n *= 10).

^1^Levels of significance: NS = *P *> 0.05, ** = *P *< 0.01, *** *P *< 0.001. ^a, b, c^Within a row, least squares means without a common superscript differ (*P <*0.05).

### Granulocyte-positive cells

#### Surface expression of L-selectin (CD62L) and neutrophil functional activity

There were no effects of treatment or treatment × sampling time interactions for neutrophil CD62L MFI or for percentage of neutrophils performing phagocytosis, but sampling time was significant (*P *< 0.05) (Table 4). On d 2, neutrophil CD62L MFI (*P *< 0.001) and percentage neutrophils performing phagocytosis (*P *< 0.05) decreased in both treatments, and subsequently did not differ (*P *> 0.05) compared with pre-weaning baseline. There was no effect (*P *> 0.05) of treatment, sampling time or treatment × sampling time interaction for the percentage and mean fluorescent intensity of neutrophils performing phagocytosis or oxidative burst activity (Table 4).

**Table 4:** Effect of concentrate supplementation for 26 d pre-weaning on neutrophil surface expression of CD62L, and neutrophil phagocytic and oxidative burst activity in abruptly weaned and housed beef calves


		**Days post-weaning**					***P*-values^1^**		
		
**Variable**		**0**	**2**	**7**	**14**	**Pooled s.e.m**.	**T**	**S**	**T×S**

**CD62L^+2^, MFI^3^**	CS	35.2^a^	20.19^b^	35.6^a^	35.2^a^	2.24	NS	*	NS
	NCS	36.2^a^	17.3^b^	36.0^a^	34.0^a^				
**Phagocytosis, %**	CS	81.1^a^	71.2^b^	78.0^a^	83.2^a^	1.99	NS	*	NS
	NCS	80.5^a^	73.2^b^	74.8^a^	80.0^a^				
**Phagocytosis, MFI^3^**	CS	12.9	10.7	16.8	14.3	0.97	NS	NS	NS
	NCS	14.7	12.9	14.8	13.01				
**Oxidative burst, %**	CS	26.0	29.4	33.1	27.3	2.34	NS	NS	NS
	NCS	24.2	23.9	26.4	25.0				
**Oxidative burst, MFI^3^**	CS	13.0	10.7	16.8	14.3	0.71	NS	NS	NS
	NCS	14.8	12.9	14.8	13.0				

T = treatment, S = sampling time, T × S = treatment × sampling time interaction.

CS = concentrate supplement prior to abrupt weaning (*n *= 10). NCS = no concentrate supplement prior to abrupt weaning (*n *= 10).

^1^Levels of significance: NS = *P *> 0.05, * = *P *< 0.05, ** = *P *< 0.01, *** = *P *< 0.001. ^a, b, c^Within a row, least squares means without a common superscript differ (*P <*0.05).

^2^CD62L = L-selectin marker.

^3^Mean fluorescence intensity = MFI.

### Lymphocyte immunophenotypes

**Figure 2:**
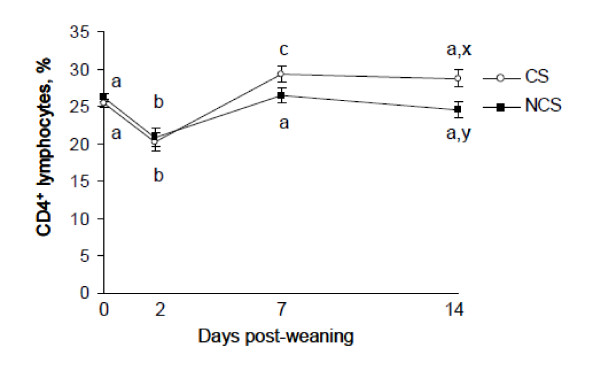
**Effect of concentrate supplementation for 26 d pre-weaning on percentage CD4^+ ^lymphocytes in abruptly weaned and housed beef calves**. CS = concentrate supplement prior to abrupt weaning (*n *= 10), NCS = no concentrate supplement prior to abrupt weaning (*n *= 10). ^a, b, c^Between days, least squares means without a common superscript differ (*P <*0.05). ^x, y^Within a day, least squares means without a common superscript differ (*P <*0.01).

There was a treatment × sampling time interaction (*P *< 0.05) for percentage CD4^+ ^lymphocytes (Figure 2). On d 2, percentage CD4^+ ^lymphocytes decreased (*P *< 0.001) in both treatments. Subsequently on d 7, percentage of CD4^+ ^lymphocytes increased (*P *< 0.01) in CS compared with d 0, whereas percentage of CD4^+ ^lymphocytes in NCS did not differ (*P *> 0.05) from d 0. There was no effect (*P *> 0.05) of treatment, sampling time or treatment × sampling time interaction for percentage CD8^+ ^lymphocytes and the CD4: CD8 ratio (data not shown).

**Figure 3:**
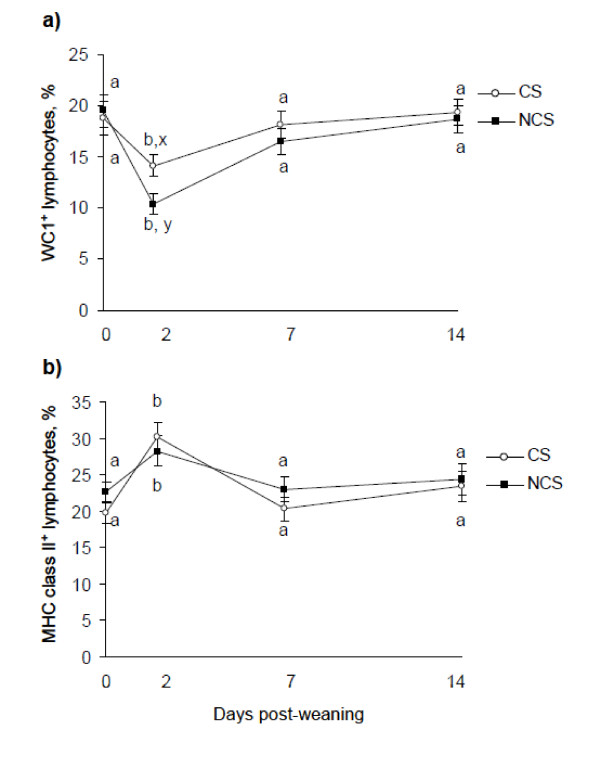
**Effect of concentrate supplementation for 26 d pre-weaning on percentage (a) WC1^+ ^and (b) MHC Class II^+ ^lymphocytes in abruptly weaned and housed beef calves**. CS = concentrate supplement prior to abrupt weaning (*n *= 10). NCS = no concentrate supplement prior to abrupt weaning (*n *= 10). ^a, b,^Between days, least squares means without a common superscript differ (*P <*0.001). ^x, y^Within a day, least squares means without a common superscript differ (*P <*0.001). There was a significant treatment × sampling time interaction for percentage WC1^+ ^lymphocytes.

There was a treatment × sampling time interaction (*P *< 0.02) for percentage WC1^+ ^lymphocytes (Figure 3a). On d 2, WC1^+ ^lymphocytes decreased (*P *< 0.05) in both treatments but the decrease was greater (*P <*0.05) in NCS than CS. Subsequently, percentages did not differ (*P *> 0.05) from pre-weaning baseline. There was no effect (*P *> 0.05) of treatment or treatment × sampling time interaction for percentage MHC class II^+ ^cells. However, on d 2, percentage MHC class II^+ ^lymphocytes increased (*P *< 0.01) in both treatments, and subsequently returned (*P *> 0.05) to pre-weaning baseline (Figure 3b).

### Acute phase proteins

There was no effect (*P *> 0.05) of treatment or treatment × sampling time interactions for concentration of fibrinogen and haptoglobin or sampling time (*P *> 0.05) for concentration of fibrinogen (Table 5). However, concentration of haptoglobin increased (*P *< 0.05) on d 2 in both treatments compared with pre-weaning baseline.

**Table 5:** Effect of concentrate supplementation pre-weaning on concentration of plasma fibrinogen and haptoglobin in abruptly weaned and housed beef calves


		**Days post-weaning**					***P*-values^1^**		
		
**Acute phase protein**		**0**	**2**	**7**	**14**	**Pooled s.e.m**.	**T**	**S**	**T×S**

**Haptoglobin, mg/mL**	**CS**	0.38^a^	0.62^b^	0.48^ab^	0.47^ab^	0.068	NS	*	NS
	**NCS**	0.35^a^	0.80^b^	0.67^ab^	0.42^ab^				
**Fibrinogen, mg/dL**	**CS**	379	456	495	432	24.9	NS	NS	NS
	**NCS**	439	524	545	486				

T = treatment, S = sampling time, T × S = treatment × sampling time interaction.

^1^Levels of significance: NS = *P *> 0.05. * = *P *< 0.05. ^a, b^Within a row, least squares means without a common superscript differ (*P <*0.05).

^2^PHA = Phytohaemagglutinin.

^3^ConA = Concanavalin A.

CS = concentrate supplement prior to abrupt weaning (*n *= 10). NCS = no concentrate supplement prior to abrupt weaning (*n *= 10).

### Metabolic responses

There was no effect (*P >*0.05) of treatment or treatment × sampling time interactions for concentrations of creatine kinase, urea, glucose, BHB and NEFA, or sampling time for concentrations of creatine kinase or glucose (Table 6). There was a treatment × sampling time interaction for globulin whereby on d 2, the increase in concentration was greater (*P *< 0.05) in CS compared with NCS, and subsequently there was no difference between treatments. Concentration of total protein was greater (*P *< 0.05) and albumin tended (*P <*0.06) to be greater in CS compared NCS. On d 2 and 7, concentration of albumin increased (*P *< 0.05) in both treatments, and subsequently did not differ (*P *> 0.05) compared with pre-weaning baseline. Concentration of urea decreased and NEFA increased (*P *< 0.05) post-weaning compared with pre-weaning baseline. On d 2, concentration of NEFA increased (*P *< 0.05), and subsequently decreased on d 14 (*P *< 0.05) compared with pre-weaning.

**Table 6:** Effect of concentrate supplementation for 26 d pre-weaning on plasma metabolites in abruptly weaned and housed beef calves


		**Days post-weaning**					***P*-values^1^**		
		
**Metabolite**		**0**	**2**	**7**	**14**	**Pooled s.e.m**.	**T**	**S**	**T×S**

**Total protein, g/L**	CS	70.3^a^	73.9^b, x^	72.8^a^	70.6^a^	0.98	*	†	NS
	NCS	68.3^a^	70.9^b.y^	72.0^b^	67.6^a^				
**Albumin, g/L**	CS	34.0^a^	33.9^a^	35.4^a^	31.6^b^	0.45	†	*	NS
	NCS	33.2^a^	33.4^a^	33.4^a^	31.0^b^				
**Globulin**,** g/L**	CS	36.6^a^	38.9^b, x^	37.7^a^	38.9^b^	0.80	NS	**	*
	NCS	35.1^a^	37.6^b, y^	38.4^b^	36.6^a^				
**Creatine kinase**,**U/L**	CS	295	304	359	317	42.7	NS	NS	NS
	NCS	307	297	324	350				
**Urea, mmol/L**	CS	4.2^a^	3.1^b^	3.1^b^	2.5^c^	0.17	NS	***	NS
	NCS	4.3^a^	3.3^b^	3.0^b^	2.5^c^				
**Glucose, mmol/L**	CS	4.5	4.6	4.3	4.3	0.08	NS	NS	NS
	NCS	4.5	4.8	4.3	4.3				
**βHB^2^, mmol/L**	CS	0.19^a^	0.27^b^	0.25^a^	0.30^b^	0.014	NS	*	NS
	NCS	0.17^a^	0.24^b^	0.23^a^	0.28^c^				
**NEFA^3^, mmol/L**	CS	0.17	0.24	0.17	0.13	0.023	NS	*	NS
	NCS	0.13^a^	0.33^b^	0.20^ab^	0.09^a^				

^1^Levels of significance: NS = *P *> 0.05, † = *P *= 0.06, * = *P *< 0.05, ** = *P *< 0.01, *** = *P *< 0.001. ^a, b, c^Within a row, least squares means without a common superscript differ (*P <*0.05). ^x, y^Within a column (day) for each variable, least squares means without a common superscript differ (*P <*0.05). T = treatment, S = sampling time, T × S = treatment × sampling time interaction. ^2^βHB = β-hydroxybutyrate, ^3^NEFA = non-esterified fatty acids.

CS = concentrate supplement prior to abrupt weaning (*n *= 10), NCS = no concentrate supplement prior to abrupt weaning (*n *= 10).

### Behavioural responses

#### Lying and standing behaviour

There was no difference between treatments for percentage of time spent lying and active and number of steps taken from d 8 to d 14. There was a treatment × day interaction (*P *< 0.001) for lying behaviour, whereby on d 0, 2, and 5, CS spent 8% (*P *< 0.01, 1 h 55 min), 9% (*P *= 0.002, 2 h 10 min) and 3% (*P *< 0.05, 43 min) more time lying, respectively, (and consequently, less time standing), compared with NCS. Time spent lying was higher (*P *< 0.001) in both treatments on d 1 to 7 (NSC: 54.0% d, 12 h 58 min, CS: 56.9% d, 13 h 40 min) compared with d 0 (NCS: 28.8 (1.78)% of d 0, 6 h 54 min; CS: 36.9 (1.78)% of d 0, 9 h 51 min). There was no effect (*P *> 0.05) of treatment or treatment × sampling time interaction for percentage of time spent active or number of steps taken per day post-weaning. However, time spent active decreased (*P *< 0.001) for both treatments by 13% and 28% on d 1 and 2, respectively, and remained decreased (*P *< 0.05) up to d 7 compared with d 0. Similarly, the number of steps taken decreased (*P *< 0.001) by 37% and 58%, on days 1 and 2, respectively, compared with d 0.

#### Feeding behaviour

There was no effect (*P *> 0.05) of treatment or treatment × sampling time interaction for the percentage time spent at the silage feed face or concentrates trough post-weaning. Sampling time was significant for percentage time spent at the silage feeding point (*P *< 0.05) whereby calves spent more time at the silage feeding point on d 7 (13.7 (1.43)%, 3 h 17 min) and 14 (15.3 (1.43)%, 3 h 40 min) compared with d 0 (NCS: 7.7 (1.42)%, 1 h 49 min; CS: 11.13 (1.42)%, 2 h 48 min).

#### Calf performance

Pre-weaning ADG for the period between d -26 and 0 was 1.07 (s.e.m.) (0.264) kg and 0.99 (s.e.m.) (0.264) kg for CS and NCS, respectively.

## Discussion

In the present study, the effect of concentrate supplementation pre-weaning on the peripheral distribution of leukocytes, the functional activity of neutrophils and the acute phase response in abruptly weaned and housed beef calves was investigated. Many factors centred on the time of weaning may heighten the susceptibility of calves to bovine respiratory disease (BRD) or may exacerbate its outcome [14]. The ability of calves to cope with weaning stress may affect their subsequent health and performance thus, reducing the negative impact of weaning stress through the use of management strategies designed to optimize their health and welfare are important considerations. Feeding concentrates is often advocated as a means of reducing weaning stress in calves through familiarisation to a palatable feed.

In the present study, the combined effect of weaning and housing resulted in neutrophilia and concurrent lymphopaenia, which is in agreement with other studies [11,12,15]. Provision of concentrates pre-weaning did not result in discernible differences in neutrophil number between treatments as it increased by approximately 50% post-weaning in both treatments. This increase is less than the magnitude of neutrophilia observed (164% increase) previously [15] in calves that were not supplemented with concentrates either pre- or post-weaning. Previous findings have indicated that increased total leukocyte number was represented by profound neutrophilia [15]. In the present study, the total leukocyte number was unchanged, and this was related to a less marked neutrophilia response. In line with previous research [15], abrupt weaning and housing decreased the percentage of neutrophils performing phagocytosis and did not affect neutrophil oxidative burst activity, and moreover, the provision of concentrates pre-weaning did not affect these functions.

In the present study, examination of lymphocyte subsets revealed that calves offered concentrates pre-weaning displayed a lesser reduction in percentage WC1^+ ^lymphocytes compared with those not supplemented. This reduction in percentage WC1^+ ^lymphocytes was also less than that obtained previously [15] where calves were not offered concentrates either pre- or post-weaning. The present findings are in agreement with other studies that have shown that γδ T cells are most sensitive to physiological and pharmacological stressors with reductions in percentage WC1+ lymphocytes observed following weaning of beef calves [15], transportation of beef steers [26], parturition in dairy cows [27], and dexamethasone treatment in dairy and beef bulls [21,28].

Additionally, calves that were offered concentrates pre-weaning had unaltered percentage of CD8^+ ^lymphocytes and increased percentage CD4^+ ^lymphocytes post-weaning compared with their non-supplemented counterparts. These findings suggest that offering concentrates may provide some level of immune protection, manifested as a lesser reduction in WC1+ (γδ T) cells and more stable αβ T cell population. An overall increase in CD4+: CD8+ ratio was reported by Degabriele and Fell (2001) [29] in sheep that were housed either in isolation or in groups. These authors attributed the rise in CD4+:CD8+ ratio to recovery of immune compensation following perturbation to lymphocyte subsets due to a stressful change in environment. Thus, CD4+:CD8+ ratio may provide more information on the recovery of homeostasis rather than occurrence of immunosuppression. Collectively, this may prove beneficial for calf health immediately post-weaning, particularly when pathogen exposure is heightened after social mixing and regrouping of unfamiliar calves during transportation and marketing as in commercial practice. Monocyte and eosinophil number were not altered by offering concentrates pre-weaning or by the combined husbandry practices of weaning and housing, which is in accordance with previous work [15]. Alterations in haematological variables reported in this study were found to be within the normal physiological range for calves of this age, and thus no negative effects on animal welfare were determined using these measures. Furthermore, the difference in rectal body temperature between supplemented and non-supplemented calves was not of clinical significance [30].

In the present study, significant differences were not realised in acute phase protein response between supplemented and non-supplemented calves, concentration of haptoglobin increased post-weaning, in line with other studies [15,31]. Hickey *et al*. (2003) [11] reported increased concentrations of fibrinogen in non-supplemented abruptly weaned bull calves.

Metabolic responses can provide valuable information on the nutritional status of an animal and can inform on global deficiencies and malnutrition. The diet of the calves pre-weaning was not restricted in either treatment as they had free access to their dams for suckling and grass herbage was freely available. As there is a period of dietary adaptation following weaning, whereby the calf ceases milk consumption and consumption of solids changes (from grazed pasture to grass silage (and concentrates)), examination of metabolic profiles is confounded by this. Hence, it can be difficult to conclude definitively on effects of stress on metabolic responses. Calves that were supplemented with concentrates had greater concentrations of total protein compared with non-supplemented calves prior to- and immediately post-weaning. Although differences in feeding behaviour, as time spent at the silage feed face or concentrate trough, did not differ between treatments post-weaning, because feed intake was not measured, it is difficult to directly attribute the increased concentration of total protein to increased intake. Increased concentration of total protein has been reported in abruptly weaned beef calves [32], however in that study animals were fasted for 24 h post-weaning, and the authors suggested that dehydration may have contributed to the elevated concentrations of total protein. Activation of the stress response is an energy-dependent process [33]. However, due to aforementioned issues it is difficult to say whether increases in energy-related metabolites (glucose, NEFA and βHB) are attributable to weaning stress or adaptation to a new diet. It is most likely that both these factors contributed to the response observed. Further research is required in disentangle these elements and to provide additional information on the dietary adaptation of weaned beef calves. Consistent with the profile described by Boland *et al*. (2008) [19], NEFA increased initially and subsequently decreased in abruptly weaned beef calves. Elevated NEFA and βHB represent a shift in energy balance in cattle, and may suggest a greater mobilisation of energy reserves in weaning-stressed calves. Elevated concentrations of NEFA and βHB have been associated with reduced neutrophil function *ex vivo *[34] in dairy cattle under negative energy balance, and this may contribute to the reduced phagocytic capacity of neutrophils post-weaning in the present study.

In agreement with other studies [18,19], on the day of abrupt weaning, calves in the present study spent approximately 32% of their time resting. Following the day of abrupt weaning, calves spent approximately twice as much time lying, which is consistent with Enríquez *et al*. (2010) [10] but contrary to Boland *et al*. (2008) [19]. The discrepancies between studies may be attributed to differences in space allowance. Where excessive space is allocated, weaned calves spend more time active and less time resting post-weaning [19]. Calves in the present study and those used by Enriquez *et al*. (2010) [10] were restricted to a space allowance of 3.7 m^2 ^per animal in slatted floor pens and 2.1 m^2 ^per animal in a corral, respectively, compared with large open space paddocks [19].

In the present study, the CS calves began to consume their full offered amount of concentrates (1 kg/day) by d -19 of the study. Feeding behaviour did not differ between treatments post-weaning with both groups spending similar percentage time at the silage feed face and concentrate trough. Although, calves that were not offered concentrates pre-weaning were initially slower to spend time at the concentrate trough on d 1, this difference was short lived and was not evident by d 2, with both groups fully adapted to the provision of concentrates in a feeding trough by d 7 post-weaning. Walker *et al*. (2007) [35] reported that paddock-weaned calves that were introduced to a feedlot were slower to find the feed bunk than their yard-weaned counterparts, however, similar to the present study, this difference in feeding behaviour was short-lived, persisting for only a few days and had no overall effect on performance. Moreover, Fell *et al*. (1998) [36] reported less morbidity in yard-weaned calves compared with pasture-weaned calves following entry into a commercial feedlot. Compared with beef calves that were weaned in paddocks, yard-weaned calves showed increased social interactions with their peers post-weaning [36].

In the present study, calves offered concentrates had a 0.09 kg numerically higher average daily live weight gain for the 26 d pre-weaning period compared with non-supplemented calves. Other authors have shown increased performance responses to *ad libitum *creep feeds with advancing days of supplementation [37], however, this is in contrast to the present study where calves received 1 kg/head daily pre-weaning. The relatively poor growth response to concentrate supplementation in comparison with other studies [4,5] may be partly attributed to factors including milk yield of the cow, herbage allowance and nutritive value and, level of concentrate supplementation.

## Conclusions

In conclusion, compared with calves that were not offered concentrates pre-weaning, those offered concentrates for 26 d prior to weaning had a lesser reduction in percentage WC1^+ ^lymphocytes and increased percentage CD4^+ ^lymphocytes post-weaning. In terms of immunocompetence, this lymphocyte subset profile may confer enhanced resistance to weaning stress. Additionally, calves supplemented with concentrate spent more time resting post-weaning compared with non-supplemented calves.

## Authors' contributions

BE and MMcG designed the study. EML and BE performed the experiments and analysed the data. EML prepared the manuscript and all authors contributed to, read and approved the final manuscript.
